# Bioactive Compounds from Leaf Vegetables as Preservatives

**DOI:** 10.3390/foods12030637

**Published:** 2023-02-02

**Authors:** Mirian Pateiro, Rubén Domínguez, Paulo E. S. Munekata, Gema Nieto, Sneh Punia Bangar, Kuldeep Dhama, José M. Lorenzo

**Affiliations:** 1 Centro Tecnológico de la Carne de Galicia, Avd. Galicia n° 4, Parque Tecnológico de Galicia, San Cibrao das Viñas, 32900 Ourense, Spain; 2 Department of Food Technology, Nutrition and Food Science, Veterinary Faculty, University of Murcia, Campus Mare Nostrum, 30071 Espinardo, Spain; 3 Department of Food, Nutrition and Packaging Sciences, Clemson University, Clemson, SC 29631, USA; 4 Division of Pathology, ICAR-Indian Veterinary Research Institute (IVRI), Bareilly 243122, India; 5 Area de Tecnoloxía dos Alimentos, Facultade de Ciencias, Universidade de Vigo, 32004 Ourense, Spain

**Keywords:** polyphenolic compounds, anthocyanins, glucosinolates, nitrate sources, antioxidant, antimicrobial, oxidative stability, shelf life, clean label, functional products

## Abstract

Trends toward a healthier diet are increasing attention to clean-label products. This has led to the search for new ingredients that avoid the use of chemical additives. Food industries are responding to these demands by incorporating natural preservatives into their products, which consumers perceive as healthy. Leafy vegetables would fit this strategy since they are common components of the diet and are associated with beneficial health effects. The objective of this chapter is to offer an overview of the large number of bioactive compounds (phenolic acids, flavonoids, anthocyanins, glucosinolates, and sulfur compounds) present in these plants, which would be responsible for their activity as potential preservatives. Its incorporation into food would improve the quality and extend the shelf life by reducing oxidative processes and inhibiting or retarding the microbial growth that occurs during processing and storage without reducing the organoleptic characteristics of the product.

## 1. Introduction

Vegetables are a very wide variety of plants that are excellent sources of nutrients and phytochemicals [1]. The main edible parts of this food group can be divided into root, stem, leaf, immature flower bud, and fruit. Leafy vegetables, also known as "greens," "vegetable greens," or "leafy greens," provide a complex profile of bioactive compounds (BACs). Although they are found in small quantities, these metabolites have an important role in the secondary metabolism of vegetables [2]. In addition, they have numerous health and nutraceutical benefits, generating high demand from consumers increasingly concerned about their well-being. In fact, the consumption of vegetables has increased significantly in recent years.

**Figure 1:**
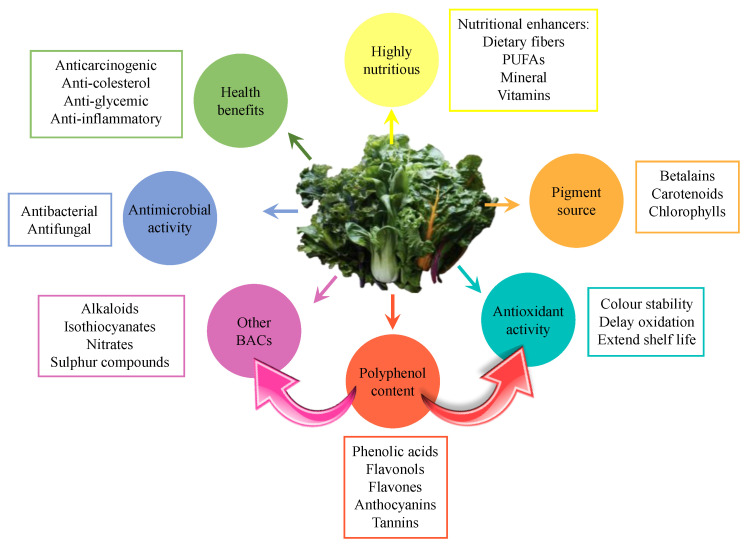
Nutritional, physicochemical properties and bioactivity associated to leaf vegetables.

The most common BACs identified in edible leafy vegetables are dietary fiber, minerals, phenolic compounds, phytic acid, phytoestrogens, polyunsaturated fatty acids (PUFAs), sulfur compounds, terpene derivatives, and vitamins [2]. Many of these BACs are antioxidant molecules with potential applications mainly in the food and nutraceutical industries (Figure 1). This is the case of polyphenols, whose ability to scavenge free radicals makes them also associated with numerous health benefits (e.g., anticarcinogenic, anti-cholesterol, anti-inflammatory, anti-glycemic, antimicrobial, and antimutagenic properties) [1]. The antioxidant activity of these compounds is mainly associated with their ability to neutralize highly reactive oxygen species (ROS) and reduce oxidation processes [3]. For all these reasons, the natural antioxidants obtained from leaves could replace the use of synthetic compounds, since some of them are even more effective and are not associated with adverse reactions.

## 2. Leaf Vegetables as a Source of Bioactive Compounds

Vegetables are excellent sources of BACs. The composition and concentration of these phytochemicals depend on the part of the plant from which they were obtained (fruits, peels, seeds, stems, or leaves). Research studies demonstrated that leaves generally have a greater variety and concentration of these BACS than those obtained from other parts such as fruits or stems [4,5]. Polyphenols represent the main class of secondary metabolites present in the leaves of vegetables, which are grouped into flavonoids and phenolic acids [6]. The information shown in the literature reflects that the main phenolic compounds identified are chlorogenic acid and quercetin, followed by ferulic acid, kaempferol, and caffeic acid [2]. The content and composition of these phytochemicals extracted from leafy vegetables depend on several factors, mainly geographical origin, genotype, physiological stage, growing conditions, and management practices [7,8]. Along with these parameters, the methods and conditions of extraction also play a crucial role in the quality and effectiveness of the obtained extracts [9,10].

The polyphenol profiles of some of the most common leafy vegetables are detailed below, including beet, bunching onion, cabbage, cardoon, cauliflower, celery, chives, dill, kale, kohlrabi, leek, lettuce, mizuna, radish, spinach, Swiss chard, and turnip.

### 2.1. Amaryllidaceae Family

**Figure 2:**
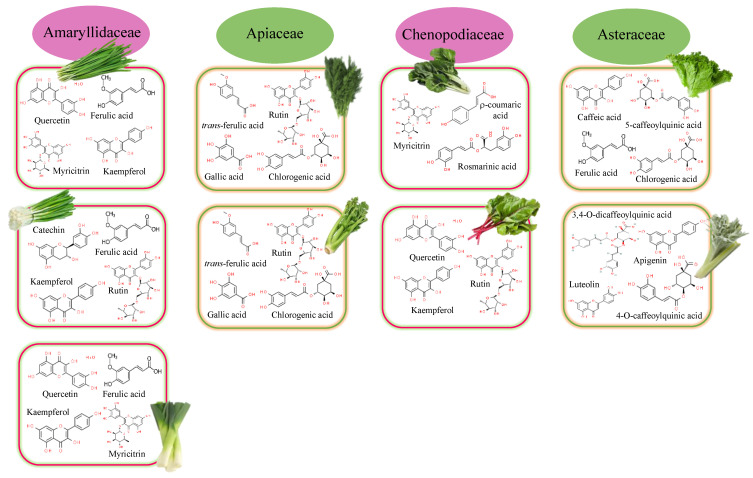
Major polyphenolic compounds found in the main families of leafy vegetables.

Allium sp. vegetables are widely consumed for their flavor characteristics but also for their antioxidant potency associated with the contents of flavonoids, specifically flavonols (kaempferol, quercetin, quercitol, isoquercetin, rutin, and myricetin), flavanones (naringenin), and flavones (apigenin, luteolin) [11,12]. Phenolic acids such as r-coumaric, ferulic, and sinapic were also commonly detected (Figure 2).

The leek plant, native to temperate regions of Europe and western Asia, is a vegetable of the species *Allium ampeloprasum*. The bulb and the leaves closest to it are considered its edible parts. The ethanolic extract of dehydrated leek leaves showed TPC values of 262.7 mg GAE/g DW, which are associated with intermediate antioxidant activity (48.6% DPPH inhibition) [13]. Ferulic acid (1.6 mg/100 g of fresh matter (FM)) and quercetin (0.8 mg/100 g FM) were the main compounds identified in its aqueous extract [14], followed by flavonoids such as myricetin (0.32 mg/100 g FM), kaempferol (0.30 mg/100 g FM), catechin (0.24 mg/100 g FM), phenolic acids such as coumaric acid (0.29 mg/100 g FM), and caffeic acid (0.09 mg/100 g FM).

Important bioactive compounds such as anthocyanins, carotenoids, flavonoids, phenols, and tannins are also part of the composition of chives (*Allium schoenoprasum*), whose chopped leaves are commonly used as an aromatic herb. Leaves and roots presented similar TPC values (52.65 and 53.52 mg/100 g, respectively), while stalk and leaf had similar free radical-scavenging capacities (10.43 and 13.71%, respectively) [15]. Beretta et al. [14] characterized the phenolic profile of the aqueous extracts, identifying high contents of myricetin (18.0 mg/100 g FM), kaempferol (15.2 mg/100 g FM), and ferulic acid (10.6 mg/100 g FM). Next in importance, quercetin (3.9 mg/100 g FM), catechin (1.4 mg/100 g FM), rutin (1.4 mg/100 g FM), chlorogenic (1.1 mg/100 g FM), coumaric acid (0.40 mg/100 g FM), and caffeic acid (0.18 mg/100 g FM) were detected. When methanol was used to obtain the extract, gallic acid was also isolated [16].

Bunching onion (*Allium fistulosum* L.) is also an interesting source of antioxidant compounds. Rutin (8.7 mg/100 g FM), kaempferol (2.0 mg/100 g FM), ferulic acid (0.80 mg/100 g FM), catechin (0.44 mg/100 g FM), chlorogenic acid (0.31 mg/100 g FM), coumaric acid (0.20 mg/100 g FM), and quercetin (0.19 mg/100 g FM) have been identified in its leaves [14].

### 2.2. Apiaceae Family

Apiaceae plants are a potential source of polyphenolic compounds [17]. *Anethum graveolens* and *Apium graveolens* are included in this family. High contents of polyphenols (2755 µg/g) were found in the leaves of *Apium graveolens* (commonly called celery), which were rich in chlorogenic acid (1790 µg/g), trans-ferulic acid (709.5 µg/g), gallic acid (73.4 µg/g), rutin (69.6 µg/g), and resveratrol (32.6 µg/g). A similar profile was observed by Liu et al. [18] in the crude extract of *Apium graveolens* L. var. dulce, where chlorogenic acid, luteolin, apigenin, and chrysoeriol were also identified. These phytochemicals are responsible for the biological activity (anti-inflammatory, antimicrobial, antinociceptive, and antioxidant) of celery leaves [19]. In the case of dill (*Anethum graveolens*), the same phenolic acids were found, although at a lower concentration (501, 96.6, and 38.8 µg/g for chlorogenic, gallic, and trans-ferulic acids, respectively). Within flavonoids, rutin (63.2 µg/g), epicatechin (41.9 µg/g) and resveratrol (16.2 µg/g) are the majority. 

### 2.3. Asteraceae Family

Lettuce (*Lactuca sativa* L.), belonging to the family Asteraceae, is one of the most popular leafy vegetables used in salads and is rich in phenolic compounds (68–212 mg/kg dry weight) [20]. Regarding the content of total polyphenols, there is great variability between the varieties of lettuce, ranging from 8.40 mg gallic acid equivalents (GAE)/100 g FW found in iceberg lettuce to 296 mg GAE/100 g FW of Lollo Rosso lettuce [21]. More than 100 compounds have been identified in their hydromethanolic extracts [22], with phenolic acids, flavonols, flavones, and anthocyanins (only in red varieties) being the most common. In general, phenolic acid was the family detected at the highest concentration, with chlorogenic acid being the most abundant (9–126 mg/kg DM), followed by caffeic acid (5–6 mg/kg DM) and ferulic acid (1–2 mg/kg DM) [23]. Hydroxycinnamic and hydroxybenzoic acid derivatives, such as caffeoyl, ρ-coumaroyl, feruloyl, dihydrocaffeoyl, sinapoyl, 4-hydroxybenzoyl, 3,4-dihydroxybenzoyl, galloyl, syringoyl, and 4-hydroxyphenylacetoyl, are also detected [22]. Caffeic acid derivatives were the main compounds identified, with 2,3-dicaffeoyltartaric acid (2,3-diCTA) and 5-caffeoylquinic acid (5-OCQA) being the major compounds detected (5.73 and 2.54 mg/g DM for 2,3-diCTA and 5-OCQA, respectively). Another class of caffeic acid derivatives, such as caffeoyltartaric acid (CTA), caffeoylmalic acid (CMA), and 3,5-dicaffeoylquinic acid (3,5-diCQA), were also identified (0.981, 0.458, and 0.177 mg/g DM for CTA, CMA, and 3,5-diCQA, respectively). Within the flavonoid family, quercetin, kaempferol, isorhamnetin, and tamarexetin were the most abundant. Sugar-conjugated quercetin forms were also identified within the flavonoid family, with quercetin-3′-O-glucuronide (Q3GA) being the majority compound, followed by 3,5-dicaffeoylquinic acid (3,5-diCQA) and quercetin-3-O-(6″-O-malonyl)-glucoside (Q3MG). These values (1.045, 0.177, and 0.162 mg/g DM for Q3GA, 3,5-diCQA, and Q3MG, respectively) were higher than those found in other vegetables such as celery. In flavones, luteolin and apigenin are the most important. Their glycosides were also identified (apigenin-O-derivate, luteolin-C-glucoside, and isorhamnetin 3-O-glucoside) but with lower contents. Finally, a flavanone glycoside was detected. It is about eriodictyol-O-glucuronide, identified for the first time in butterhead lettuce by Viacava et al. [22].

A valuable source of phenolic compounds was also found in the leaves of wild cardoon (*Cynara cardunculus* L. var. *sylvestris* (Lamk) Fiori) [24]. More than half of the total phenolic compounds (TPC) quantified (38.3 mg/g extract) in the hydroethanolic extracts obtained from the leaf blade are phenolic acids (23.6 mg/g extract). In this family, caffeoylquinic acid derivatives showed the highest contents. Among them, 4-O-caffeoylquinic acid (13.6 mg/g extract) and 3,4-O-dicaffeoylquinic acid (6.5 mg/g extract) stand out. Within flavonoids, luteolin (12.89 mg/g extract) and apigenin (1.85 mg/g extract) derivatives were found in wild cardoon leaves. 

### 2.4. Brassicaceae Family

*Brassica* genus includes many species of great interest for their potential as a source of natural antioxidants, especially polyphenols, which contribute to the biological activity of these plants [25]. Broccoli, Brussels sprouts, cabbage, cauliflower, and kale, belonging to the genus Brassica oleracea, stand out for their high content of health-promoting phytochemicals [26]. Polyphenol content varied significantly between varieties, according to previous research [27,28].

**Figure 3:**
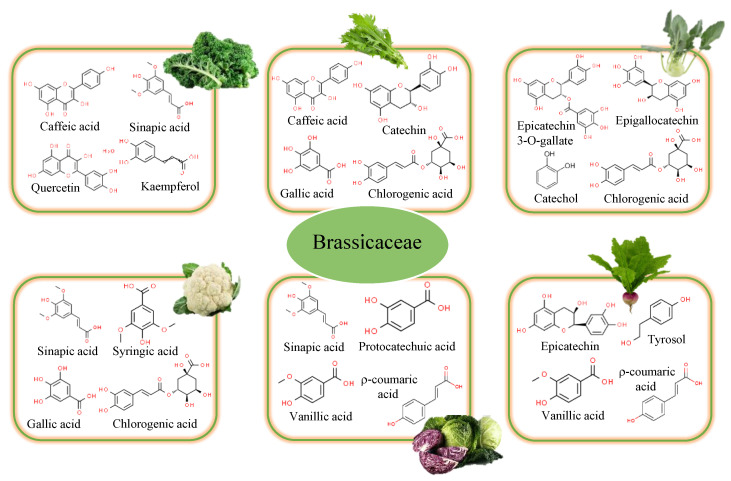
Main bioactive compounds responsible for Brassicaceae activity.

Cauliflower (*Brassica oleracea* L. var. *botrytis*) is one of the most widely consumed cruciferous vegetables in the world. Polyphenol levels in its methanolic extracts were found to be adequate (Figure 3), indicating an important antioxidant activity [29]. However, there are hardly any studies on the polyphenolic profile of its leaves. Sinapic acid (4.28 mg/100 g), syringic acid (1.13 mg/100 g), gallic acid (0.69 mg/100 g), chlorogenic acid (0.63–4.23 mg/100 g), ferulic acid (0.53 mg/100 g), protocatechuic acid (0.44 mg/100 g), and 5-CQA (0.10 mg/100 g) have been reported as the major phenolic compounds [2].

Cabbage (*Brassica oleracea* var. *capitata*) is a plant with green, red (purple), or white (pale green) leaves that belongs to the so-called “cabbage crops”. Their TPC ranged between 19.4 and 75.8 mg GAE/100 g of fresh weight (FW) [21]. In the case of white varieties, they were characterized by phenolic acid contents of 1715.69 μg/g of dry weight (DW) [30]. Jaiswal et al. [31] studied their phenolic acid contents. The results obtained reflected that they are characterized by higher contents of hydroxybenzoic acids (3.69–4.86 GAE/g) than those found for hydroxycinnamic acids (0.49–1.14 chlorogenic acid equivalents (CAE) g/L). Regarding hydroxybenzoic acids, vanillic acid (745.85 μg/g DW) was the main compound identified, followed by protocatechuic acid (70.81 μg/g DW), isovanillic acid (9.90 μg/g DW), and trans-cinnamic acid (5.99 μg/g DW). Within hydroxycinnamic acids, sinapic acid (405.62 μg/g DW), ρ-coumaric acid (304.96 μg/g DW), ferulic acid (115.30 μg/g DW), and caffeic acid (53.45 μg/g DW) were identified. These contents were lower than those found in red cabbage (*Brassica oleracea* var. *capitata* f. *rubra*), whose leaves have a characteristic purplish color. These varieties present values of 150 mg GAE/100 g, 50 mg QE/100 g, and 2.7 mg/100 g for TPC, total flavonoid contents (TFC), and total anthocyanin content, respectively [32]. The main phenolic compounds (26,881.07 μg/g DW) identified were sinapic acid (10,067.37 μg/g DW), vanillic acid (5961.13 μg/g DW), ρ-coumaric acid (4518.52 μg/g DW), protocatechuic acid (2881.17 μg/g DW), ferulic acid (2768.48 μg/g DW), and caffeic acid (650.41 μg/g DW) [30]. It is also important to note that the red variety also showed a higher content of flavonols (26.58 μg/g DW) and anthocyanins (4984.13 μg/g DW) [30]. Within flavonols, the contents of kaempferol (15.43 μg/g DW) and quercetin (9.21 μg/g DW) stand out. Upadhyay et al. [29] discovered the presence of rutin (102.14 μg/g FW). In anthocyanins, the highest contents were found for Cy-3-(Sin)-diGlu-5-Glu (852.24 μg/g DW) and Cy-3-diGlu-5-Glu (640.88 μg/g DW). Lower contents of TPC (1.25 mg GAE/g FW vs. 2.26 mg GAE/g FW) and TFC (0.71 mg RE/g FW vs. 1.43 mg RE/g FW) were also found in green cabbage by Upadhyay et al. [29] when compared to red cabbages. This was reflected in a lower antioxidant capacity (0.81 mg/g FW vs. 1.15 mg/g FW for green and red cabbage, respectively). Their phenolic profile displayed that sinapic acid (3.52 μg/g FW), gentisic acid (2.50 μg/g FW), rutin (1.34 μg/g FW), and cinnamic acid (0.65 μg/g FW) were the main compounds identified in the methanol extracts. 

Kohlrabi (*Brassica oleracea* var. *gongylodes* L.) could also be considered a rich source of phenolic acids and flavonoids. The aqueous extract from leaves showed contents of TPC, TFC, and flavonols of 5290.6 mg GAE/100 g, 718.7 mg catechin equivalents (CE)/100 g, and 479.3 mg CE/100 g, respectively [33]. The main compounds identified were epicatechin 3-O-gallate (113.5 mg/100 g DW), chlorogenic acid (106.4 mg/100 g DW), catechol (89.9 mg/100 g DW), and epigallocatechin (82.2 mg/100 g DW). A greater number of compounds were detected when methanol was the solvent used to carry out the extraction, probably due to its polarity, which plays a crucial role in increasing the solubility of phenols [34]. Hydroxycinnamic acids (chlorogenic, r-coumaric, ferulic, and sinapic acids) and hydroxybenzoic acids (syringic and gallic acids) were identified in the methanolic extracts obtained from its leaves [35]. Moreover, flavonoids such as catechin hydrate, catechol, epigallocatechin, and epicatechin 3-O-gallate were also detected.

Kale (*Brassica oleracea* var. *acephala*) is an excellent source of fiber as well as vitamins and minerals. Its nutritional value qualifies it as a superfood. But it is also considered a good source of polyphenolic compounds [36]. Among its varieties, Galega kale (*Brassica oleracea* var. *acephala* cv. Galega) stands out for its high contents of phenolics (314.8–322.3 GAE mg/100 g) [37]. Caffeic acid, ferulic acid, and sinapinic acid derivatives were the main phenolic acids identified in its leaves, while quercetin and kaempferol glycosides were the predominant flavonoids [38]. However, it is necessary to emphasize that this composition varies a lot depending on variety, maturity stage, growing location, and environmental conditions [36]. In fact, other hydroxybenzoic acid derivatives such as ρ-hydroxybenzoic acid (624 ng/g), protocatechuic acid (131 ng/g), vanillic acid (115 ng/g), gallic acid, and salicylic acid (13.8 ng/g) were also identified. In the group of hydroxycinnamic acid derivatives, ρ-coumaric acid was also detected [39]. Kale varieties also contain anthocyanins, predominantly cyanidin glycosides, which in red varieties can represent up to 7.6 times more than in green kale (1.074 vs. 0.141 µmol/g dry weight, respectively) [40]. Cyanidin 3-(sinapoyl)diglucoside-5-glucoside content was identified as the most abundant (0.39 µmol/g dry weight), followed by cyanidin 3-(p-coumaroyl) diglucoside-5-glucoside (0.14 µmol/g dry weight). These cyanidins were not detected in green kale, where cyanidin 3-(p-coumaroyl)(sinapoyl)diglucoside-5-glucoside (0.068 µmol/g DW) and cyanidin 3-(feruloyl)(sinapoyl)diglucoside-5-glucoside (0.052 µmol/g DW) were the ones with the highest content.

Turnip, rapini, or mizuna are included within *Brassica rapa*. In the case of turnips, the most consumed part is the root. Although the leaves are not usually used for human consumption, only in a few countries are they often consumed as “turnip greens”. They are an important source of bioactive compounds. In this regard, Chihoub et al. [24] found TPC of 25.0 mg/g in the hydroethanolic extracts, with flavonoids being the most abundant (18.8 mg/g). In this group, fourteen flavonoids were identified, highlighting the contents of kaempferol glycosylated derivatives, followed by quercetin and isorhamnetin derivatives. Isorhamnetin-O-dihexoside (2.8 mg/g), kaempferol-3-O-sinapoylsophoroside-7-O-glucoside (1.9 mg/g), and quercetin-3-O-sophoroside (1.1 mg/g) were the most representative. In addition, hydroxycinnamic acid derivatives were also detected, with synapoylmalic acid being the most notable (2.8 mg/g). This compound would be responsible for the antioxidant, antimicrobial, anti-inflammatory, anti-cancer, and anti-anxiety activities of this vegetable [41]. Ferulic, caffeic, and ρ-coumaric acids were also identified.

Another leafy vegetable of the species *Brassica rapa* is mizuna (*Brassica rapa* L. var. *japonica*). This plant, commonly used as a salad vegetable, is rich in polyphenols. Chlorogenic acid (176.5 µmol/g DW), caffeic acid (120.1 µmol/g DW), gallic acid (1.48 µm/g DW), catechin (154.2 µmol/g DW), and (−)-epicatechin (1351.2 µmol/g DW) were the main phenolic compounds identified. The contents of gallic acid (4.71 µmol/g DW), chlorogenic acid (184.9 µmol/g DW), and catechin (234.1 µmol/g DW) were higher in the red variety [42]. In addition to these compounds, anthocyanins were also identified in red mizuna, the majority of which were cyanidins. Among them, we highlighted the contents of cyanidin 3-(sinapoyl)(sinapoyl)diglucoside-5-glucoside (0.76 µmol/g DW), responsible for the red color of these products.

Chinese cabbage (*Brassica rapa* ssp. *pekinensis*), also known as napa cabbage, is an important vegetable crop that contains several species that are of horticultural importance. In its outer leaves, four kinds of phenolic acids were identified [43]: caffeic acid (1.39 mg/100 g DW), sinapic acid (6.82 mg/100 DW), ρ-coumaric acid (2.89 mg/100 g DW), and ferulic acid (0.47 mg/100 g DW), and ferulic acid (0.47 mg/100 g DW). These hydroxycinnamic acid derivatives have also been found in other green leafy vegetables, such as lettuce [22]. Along with these compounds, the flavonoid myricetin (0.83 mg/100 g DW) was detected. In the case of pak choi (*Brassica rapa* subsp. *chinensis*), different profiles have been found in white, pale green, and green cultivars (362.67, 799.88, and 795.30 and μg/g DW, respectively). In all cultivars, ferulic acid (252.16, 451.25, and 393.53 μg/g DW for white, pale green, and green cultivars, respectively) was the predominant phenolic acid. Whereas in the flavonoid family, differences were found among samples [44]. Rutin was the majority in white pak choi (15.45 μg/g DW), while epicatechin was predominant in the other two cultivars (138.13 and 168.86 μg/g DW for pale green and green cultivars, respectively).

The leaves of radish (*Raphanus sativus* L.) are considered an underutilized leafy vegetable of the *Cruciferaceae* family [45]. However, their TPC is very high, doubling the contents found in the root (695.1 vs. 341.5 mg GAE/100 g DM) [10]. Higher contents (1455 mg GAE/100 g DM) were obtained when supercritical CO_2_ extraction was applied at 40 °C/400 bar to extract BACs from radish leaves [46]. This could be related to the fact that the plant’s extraction efficiency is highly dependent on the extraction technique and its operating conditions [9]. In the case of flavonoids, the contents get to quadruple those obtained from the root (1042.7 vs. 267.5 mg quercetin equivalent (QE)/100 g DM). These values were lower than those found using emerging technologies (2180 mg QE/100 g DM) [46]. Specifically, epicatechin (322.1 mg/100 g DM), tyrosol (180.5 mg/100 g DM), vanillic acid (156.0 mg/100 g DM), ρ-coumaric acid (43.5 mg/100 g DM), trans-sinapic acid (34.0 mg/100 g DM), caffeic acid (27.3 mg/100 g DM), and trans-ferulic acid (7.60 mg/100 g DM) were the most abundant compounds identified in the leaves [10]. Important contents of kaempferol derivatives were also found by Chihoub et al. [24] in hydroethanolic extracts obtained from the aerial tops (leaves and stems) of *Raphanus sativus* L., where citric acid was also detected.

### 2.5. Chenopodiaceae Family

Swiss chard, beets, and spinach are included within the *Chenopodiaceae* family. Their leaves can be consumed in salads or cooked alone or along with the stems. Swiss chard (*Beta vulgaris* L.) is a potential source of polyphenolic compounds, where *Beta vulgaris* L. var. cicla is the most studied variety. Mzoughi et al. [47] calculated the phenolic composition of the ethanolic extract obtained from its leaves. The authors found values of 96.58 mg GAE/g DW for TPC, 30.08 mg of CE/g for TFC, 22.69 mg of rutin equivalents (RE)/g for total flavonol content, 41.80 mg CE/g for total tannin content, and 7.66 mg of hydroxytyrosol equivalents (HE)/g for total orthodiphenol content. The high contents found for TPC (>20 mg/g) make it a promising natural antioxidant [48]. Regarding phenolic acids, myricitrin (4.08 mg/g extract), ρ-coumaric (3.53 mg/g extract), and rosmarinic acids (1.02 mg/g extract) were the main ones identified. Within flavonoids, 2″-xylosylvitexin (193 mg/100 g FW), isorhamnetin 3-gentiobioside (39 mg/100 g FW), 6″-malonyl-2″-xylosyl vitexin (34 mg/100 g FW), and isorhamnetin 3-vicianoside (10 mg/100 g FW) were the most prominent [47].

Beet (*Beta vulgaris* subsp. *vulgaris*), also known as beetroot, is one of the varieties of *Beta vulgaris* that are cultivated for their edible roots and leaves (called beet greens). In addition to its nutritional value, it stands out among commonly consumed leafy vegetables for its BAC content, which is higher than that of other leafy vegetables, giving it a high capacity as a preservative [49]. Pellegrini and Ponce [13] found TPC values of 508.9 mg GAE/100 g DW in the ethanolic extracts obtained from its dehydrated leaves. Higher outcomes were obtained by Biondo et al. [50] in methanolic extracts of dehydrated beetroot leaves that were organically produced. These values are correlated with the antioxidant capacity (69.2% DPPH inhibition) associated with these extracts, which can be considered strong according to the classification proposed by Soquetta et al. [51]. The same values (1500 mg/100 g) were found by Fernández et al. [49]. Regarding the flavonoid profile, rutin was the predominant compound identified, with contents (9.7 mg/kg) that accounted for 61% of total polyphenols. Quercetin and kaempferol were also detected but at much lower concentrations (0.012 and 0.001 mg/kg, respectively). 

## 3. Inclusion of Leaf Vegetable Extracts in Foods

The high levels of polyphenols found in leafy vegetables ensure their effectiveness as radical scavengers, prevent the growth of pathogenic and spoilage bacteria, and provide beneficial effects that would allow consumers to improve their health. Therefore, the application of these natural preservatives in food is a promising strategy as a replacement for synthetic additives.

In this regard, several publications have focused on the use of natural preservatives extracted from vegetables in order to decrease lipid and protein oxidation, inhibit microbial growth, and improve the shelf life without negative effects on sensory attributes [9,52].

### 3.1. Bakery and Pastry Products

Leaf vegetables have been used in bakery and pastry products, such as biscuits, cereal bars, and chocolates, to improve their nutritional value and preserve their desirable attributes. The addition of functional ingredients to chocolates could improve their nutritional properties. The study conducted by Carvalho et al. [53] demonstrated that the incorporation of lyophilized kale (1% *w*/*w*) into the formulation of milk chocolate improved the nutritional profile and the transference of phenolic compounds from kale to the final product. In this regard, the incorporation of kale increased the contents of ash (2.53 vs. 2.68 g/100 g) and soluble fiber (3.18 vs. 4.18 g/100 g) compared to the values found in the control. On the contrary, fat (36.26 vs. 35.06 g/100 g) and sugar contents (21.00 vs. 17.00 g/100 g) decreased, while other components such as protein or moisture contents were not affected by the inclusion of kale. Regarding TPC, the results showed that there was no significant difference between milk chocolate and kale-enriched chocolate (74.22 vs. 74.29 mg/g GAE). However, the authors noticed the presence of benzoic acid and cinnamic acid derivatives in the polyphenol profile of kale chocolate. This confirmed the transfer of BACs from kale to chocolate, such as hydroxycinnamic acids (caffeic acid, ferulic acid, cummaric acid, and sinapic acid) and hydroxybenzoic acids (galic acid, vanilic acid). Finally, the sensory analysis results noticed that chocolates formulated with kale had lower purchase intention than those obtained in control samples (61.1% vs. 66.6%).

Galla et al. [54] evaluated the effect of supplementing biscuits with spinach (*Spinacia oleracea* L.) on nutritional, texture, and sensory quality. The incorporation into the cookie dough led to an increase in fiber, mineral, and protein contents, which were higher as the percentages of leaf powder increased. However, texture was affected by the addition of spinach, more specifically hardness and breaking strengths, which affected the overall acceptability of the product. In this regard, as the powder contents increased, there was a decrease in breaking strength, so that although it is close to the control values (90 vs. 318 g for the control and 15% spinach-based biscuits, respectively), it would compromise the acceptability (8.00 vs. 6.00). On the contrary, the lowest dose barely modified the hardness (2138 vs. 2395 g for control and biscuits supplemented with 5% spinach powder, respectively), but increased breaking strength (90 vs. 808 g for control and 5% spinach-based biscuits, respectively). Moreover, the increase in the spinach content resulted in the modification of the flavor of the product, reducing it and giving rise to the appearance of bitter flavors. Therefore, the cookies with the lowest dose of spinach were the most acceptable (7.42, 6.54, and 6.00 for biscuits treated with 5, 10, and 15% spinach powder, respectively).

Similar results were observed when broccoli leaf powder (2.5, 5, and 7.5%, *w*/*w*) was used to improve the nutraceutical potential of gluten-free mini sponge cakes [55]. Broccoli by-products, which account for approximately 30% of vegetable biomass, are distinguished not only by their high nutrient content but also by their BAC content [56,57]. In this regard, broccoli leaf powder had a high TPC (9.56 mg GAE/g DM) with multiple antioxidant properties (99.83 and 38.07 µmol trolox/g DM for ABTS and FRAP, respectively) [55]. This was reflected in the cakes that increased their TPC compared to the control in a dose-dependent manner (from 67% to 115% for samples with 2.5% and 7.5% of broccoli leaf powder, respectively). In the same way, the antioxidant capacity increased (1.15 vs. 2.57, 3.22, and 4.19 µmol trolox/g DM for ABTS and 0.24 vs. 1.04, 1.73, and 2.77 µmol trolox/g DM for FRAP). In contrast, sensory evaluation showed that broccoli leaf powder should be used in moderate amounts to preserve the desirable attributes. Thus, samples with the least amount of broccoli leaf powder were the ones that obtained the best scores, while higher doses contributed to the dark green color, hardness, intense taste, and aroma of broccoli. In contrast to the aforementioned results, the incorporation of the flour obtained from the leaves of fresh white cauliflower (*Brassica oleracea* L. var. *botrytis*) into biscuits was not satisfactory due to their limited acceptable sensory characteristics [58].

In view of the results shown above, it seems that it is still necessary to improve the products obtained since this reformulation must satisfy the nutritional demands of consumers without significantly affecting the organoleptic characteristics of traditional products.

### 3.2. Dairy Products

The shelf life of dairy products is conditioned by pathogenic and spoilage microorganisms due to the presence of favorable conditions for microbial proliferation (*Listeria monocytogenes*, *Escherichia coli*, and *Staphylococcus aureus*) [59]. Therefore, they are products that are very susceptible to contamination, which makes the use of preservatives necessary [60]. The controversy generated around the use of synthetic antioxidants is prompting trends towards the use of natural ingredients. In this regard, the use of natural preservatives derived from leafy vegetables in dairy products is a promising strategy for enhancing the nutritional value and shelf life of fermented products (cheese and yoghurt). However, in recent years, there have hardly been any studies that have evaluated the incorporation of leafy vegetables or their extracts in these types of products (Table 1), which could be related to the contribution that these products can have on the sensory attributes.

Awda et al. [61] evaluated the effect of the addition of celery (*Apium graveolens*) leaf on the microbial population and sensory properties of white soft cheese. The celery leaves, previously finely cut and soaked in water at 75 °C, were added in three doses (5, 10, and 15%) to the raw milk with which the cheese was made. The incorporation of celery leaves had no significant effect on the proximate composition. However, despite not being significant, the use of a higher concentration of celery leaves showed a lower protein concentration, lipid content, and ash content (16.81, 15.13, and 4.30%, respectively), but a higher moisture content (59.50%). Celery leaves had a preservative effect, decreasing the counts in the treated samples (8.9 vs. 1.8 × 10^5^ CFU/g for the control and samples treated with 5% celery after 15 days of storage). Moreover, total bacterial counts (TVC) were significantly affected by increased levels of celery leaves in the sample during storage. Samples treated with the highest dose displayed the lower values at the end of storage (1.3 × 10^5^ CFU/g vs. 2.9 and 6.0 × 10^5^ CFU/g for samples treated with 15, 10, and 5%, respectively). Moreover, sensory analysis revealed that there was no significant effect on overall acceptability; even white soft cheese prepared with 5% and 10% had higher scores compared to the control cheese (50, 54, and 48, respectively).

*Allium roseum* (rosy garlic), whose leaves are traditionally used as a condiment because of their specific taste, are considered a source of BACs with antibacterial, antifungal, and antioxidant properties [65]. Gliguem et al. [62] studied the effect of supplementation with *Allium roseum* leaves in powder form (0.8%) and fresh paste (6%) in double cream cheese. The results demonstrated their potential as natural preservatives, since they even improved the product quality. The antimicrobial properties of *Allium roseum* leaves were demonstrated by their ability to reduce the growth of yeasts and molds and inhibit the growth of total coliforms during storage. This could be due to the important content of alkaloids, flavonoids, phenolic acids, and sulfur compounds in their composition [66]. In this regard, some studies have shown that Allium species are able to inhibit more than 80 species of pathogenic molds. Among the aforementioned BACs, the contents of allicin (diallylthiosulfinate) stand out, to which important antibacterial effect (*Escherichia coli*, *Staphylococcus aureus*, *Salmonella enteritidis*) and antifungal properties were attributed [67]. Moreover, the addition of *Allium roseum* leaves improved the lipid stability of cheese, significantly increasing its shelf life compared to the control (12 vs. 10 days). This could be due to the presence of flavonoids in their composition, especially kaempferol and its conjugates, luteoline and apigenine, which have been associated with antioxidant properties [67]. Significant differences were also found among samples for sensory attributes. In general, treated samples displayed higher scores for all of the attributes evaluated. Specifically, cheese with 0.8% powder displayed the highest scores for pungent and spicy smells and tastes, while samples with 0.6% fresh paste had higher scores for bitter taste, granular texture, and aftertaste. Although the characteristic smell (associated with the allicin content) would be expected to influence product acceptance, cheeses flavoring *Allium roseum* paste were those that showed the best overall acceptability (7.97 vs. 6.56 and 5.10 for paste, powder, and control samples, respectively).

Positive effects of *Allium roseum* leaf powder were also observed by El Hatmi et al. [63] on the radical-scavenging activity of soft cheese made from ultrafiltered dromedary milk. The powder was characterized by TPC of 222.5 mg GAE/100 g, TFC of 205.0 mg QE/100 g and DPPH•-inhibition of 261.7 mg Trolox/100 g. The phenolic profile showed that ρ-coumaric acid (605.58 mg/100 g) was the most abundant compound, followed by trans-ferulic acid (209.95 mg/100 g) and quinic acid (83.33 mg/100 g). Apegenin (62.27 mg/100 g) and kaempferol (50.31 mg/100 g) were the predominant flavonoids identified. These results were reflected in the cheese supplemented with 0.5% (*w*/*w*), where quinic acid (from 17.73 to 26.60 μg/g), ρ-coumaric acid (from undetected levels to 6.33 μg/g), kaempferol (from undetected levels to 2.58 μg/g), apegenin (from 0.15 to 1.93 μg/g) and quercetin (from undetected levels to 0.35 μg/g) increased their contents. Regarding the effect on the physicochemical properties, enriched cheese displayed a darker color than that observed in control samples. However, these changes did not significantly alter the sensory quality of the product.

Spinach is also considered a good source of nutrients and compounds with important antioxidant properties. Therefore, it could be considered a functional ingredient for the formulation of new dairy products. El-Sayed [64] evaluated the inclusion of spinach nano-powder (0.50, 1.00, 1.50, and 2%) in the production of soft cheeses from the retentate of whole fresh UF buffalo milk. Previous analyses carried out on the powder showed a TPC of 11.63 mg/g DW and a DPPH scavenging activity of 48.58%. This was reflected in the cheese, where the TPC (60.55–110.56 mg/g) and the antioxidant activity (19.53–35.84%) increased with the spinach dose. In addition to improving the nutritional value, the inclusion of spinach powder improved the sensory attributes. On the one hand, the green color of the spinach was reflected in the cheese, which was gradually increased with the addition of spinach powder. Secondly, aroma and flavor were also affected in a dose-dependent pattern, reducing flavor and introducing bitter flavors. This resulted in greater acceptance of cheeses with small amounts of spinach powder (0.5% and 1%), which obtained higher scores (90.22 and 86.36) compared to samples treated with 1.5 and 2% (83.65 and 81.09), and similar to those observed in the control (90.79). These results corroborate those previously found by Galla et al. [54] in biscuits supplemented with spinach.

### 3.3. Fish and Fish Products

Fish and fish products are a very important source of protein and polyunsaturated fatty acids (PUFAs), which makes them a very nutritious food. However, these same characteristics make it very susceptible to deterioration [68]. In consequence, it is very important to prevent lipid oxidation and microbial spoilage in seafood products to ensure their safety and quality. As we have mentioned, leafy vegetables are natural sources of antimicrobial and antioxidant compounds, and therefore, they could be used to preserve fresh fish and its processed products (Table 2).

**Table 2:** Effect of natural extracts obtained from the leaves of different endemic plants on the shelf life of muscle foods.

Leaf Vegetable	Product	Extract Dose	Storage Conditions	Main Results	Ref.
*Allium paradoxum*	Silver carp (*Hypophthalmichthys molitrix*) fillets	2 and 4% (*w*/*v*)	15 days at 4 °C	Delayed oxidative and microbial deterioration during refrigerated storage. *A. paradoxum* allowed for increased shelf life with a positive effect on sensory quality.	[69]
Beet, lettuce, arugula, spinach, chard, celery, and watercress	Spanish chorizo	1500–3000 ppm	125 days under retail conditions	The combination of citric extract and rosemary with leafy green vegetable extracts rich in nitrates allowed for the production of a clean-label product.	[71]
Bok choy, chamnamul, chinese chives, and pumpkin	Ground beef patties	0.1% and 0.5% (*w*/*w*)	12 days at 4 °C	The application of chamnamul reduced the microbial load and delayed lipid oxidation during refrigerated storage. The extracts naturally contained a green pigment, which could have a negative impact on the color of the patties.	[72]
Cauliflower (*Brassica oleracea*)	Tuna fish burger	0, 3, 6, 9 and 12%	4 months at −18 °C	TBARS, TVB-N, and microbial load decreased by increasing the levels of DCP, without negative effects on sensorial analysis.	[73]
	Pork patties	2.5, 5.0, and 10 g/kg	9 days at 4 °C	Inhibition of lipid and protein oxidation and microbial growth in pork patties in a dose-dependent pattern. Improved sensory attributes of pork patties.	[74]
Green cabbage (*Brassica olerecea*)	Chicken meatballs	0, 15 and 25%	9 days at 4 °C	The replacement of meat by green cabbage resulted in a decrease in lipid oxidation and microbial growth, allowing for better maintenance of sensory attributes during shelf life.	[75]
	Mutton patties	6, 9 and 12%	21 days in aerobic packaging and 60 days in vacuum packaging at 4 °C	The incorporation of cabbage allowed for the production of a safe product without loss of physico-chemical, color, microbiological or sensory quality.	[76]
Green and red cabbage	Chicken croquettes	shreds (20%) aqueous extracts (7%)	One-month frozen storage	High nutritional and antioxidant potential without affecting product acceptability.	[77]
Kimchi	Ground pork meat	1 g/kg	14 days at 4 °C	Positive effect on color, displaying lower deterioration (ΔE* and MetMb). Lower TBARS values (<0.4 mg MDA/kg).	[78]
Red cabbage	Fresh minced tilapia (*Nile perch*)	17, 34, and 68 ppm	10 days at 4 °C	Less degradation of the product during its shelf life.	[79]

ΔE*, total color difference; DCP, dehydrated cauliflower powder; MDA, malonaldehyde; MetMb, metmyoglobin; TVB-N, total volatile basic nitrogen; TVC, total viable counts.

In fresh fish, Raeisi et al. [69] evaluated the effect of ethanolic extracts of *Allium paradoxum* leaves on the oxidative and quality deterioration of silver carp fillets during refrigerated storage. For that, fish fillet samples were dipped into extracts of *A. paradoxum* at 2.0 and 4.0% (*w*/*v*) and packaged in polyethylene bags. Peroxide (PV) and TBARS were used as deterioration indices of primary and secondary oxidation, respectively. The treated samples showed lower values than those observed in the control. Therefore, *A. paradoxum* ethanolic extracts were able to retard the production of lipid oxidation products, which would be related to the antioxidant properties of sulfur, limonene, and linoleic acid present in the extracts [70]. In the case of PV, treated samples maintained the values below the limits of acceptability (<20 meq O_2_/kg) for 9–15 days, while this value was reached after 6 days in the control samples. Moreover, a dose-dependent effect was observed since the effect was greater when a higher extract concentration was used. The same trend was observed in the values obtained for secondary oxidation. In the case of fillets treated with the highest dose of the extract, TBARS values stayed below the acceptable limit (<2 mg MDA/kg) until the end of storage, while this threshold was reached at 9 days in samples treated with 2% *A. paradoxum* and at 6 days in the control samples. Regarding quality deterioration indices, the BACs present in the extracts (listed above) also played a marked role in lipid oxidation stabilization. This was reflected in the outcomes obtained for the acid value, which were again lower in treated samples (7.12 vs. 7.21 mg KOH alcohol/g for 4% *A. paradoxum* and control, respectively). Finally, the extracts allowed for a delay in the formation of total volatile basic nitrogen (TVB-N) in fish samples, keeping the values under 20 mg N/100 g sample for a longer time. On the other hand, *A. paradoxum* extracts showed an antibacterial effect on TVC and Gram-negative psychrotrophic bacteria, reducing the microbial counts when these were compared to control samples. These results were completed with a sensory study, where it was reflected that the treatment with *A. paradoxum* extracts improved some sensory attributes and maintained the acceptability of the product. Therefore, *A. paradoxum* leaf extracts are a good strategy to extend the shelf life of fish.

Positive effects were also found when red cabbage extracts (17, 34, and 68 ppm) were incorporated into fresh minced tilapia (*Nile perch*) [79]. The extracts, which showed antioxidant and anthocyanin contents of 2100 μmol/g FW and 25 μg/g FW, allowed for a decrease in the oxidative processes in minced fish. All the samples displayed low TBARS values (<0.6 μmol malonaldehyde/g sample), with the samples treated with 68 ppm of red cabbage extract presenting the lowest values. Similar behavior was observed in PV values. 

In fish products, various types of cooking methods are carried out before consumption. During this processing, the stability of the functional ingredients can be modified, and the effect will depend on the procedure used. This will influence the nutritional profile and the sensory acceptability of the product [80]. Leafy vegetable powders or extracts are also a good alternative to synthetic additives in fish products. Aamer and Emara [73] tested the possibility of using dehydrated cauliflower (*Brassica oleracea* L.) powder (0, 3, 6, 9, and 12%) as a natural antioxidant and antimicrobial preservative to prepare dietary fiber-enriched fish burgers. The previously characterized dehydrated powder showed values of TPC and TFC of 756.26 and 155.83 mg/100 g, respectively, with an antioxidant activity of 54.67%. The incorporation of the dehydrated powder into the burger formulation resulted in the transference of phenolic compounds from cauliflower to the final product. This was reflected in the values of TPC, TFC, and antioxidant activity, which increased as the added levels of powder increased. In addition to improving the technological properties of the product, dehydrated cauliflower powder maintained the TBARS and TVB-N values below the limits of acceptability throughout the shelf life of the product. This effect was greater when higher doses of the extract were used. The antimicrobial effect of phenolic and flavonoid compounds in cauliflower powder was reflected in the microbial load of fish burgers. In general, the values did not exceed the maximum levels allowed in fish products. The TVC, coliform, psychrophilic bacteria, yeast, and mold counts were lower in treated samples. Unlike other products in which the addition of cauliflower led to a decrease in the sensory quality due to its intense odor and taste, in this case the addition of dehydrated powder was suitable (odor: 7.60 vs. 6.90 for 9% cauliflower powder and control, respectively; taste: 7.50 vs. 6.60 for 3% cauliflower powder and control, respectively). The scores increased with the addition of the extract and with increasing the dose used, with samples with 6% and 9% being those that presented better sensory attributes (7.50 vs. 6.70 for cauliflower powder and control, respectively). 

### 3.4. Meat and Meat Products

In the same way as fish and fish products, meat and meat products are sensitive to lipid and protein oxidations and susceptible to spoilage by foodborne microorganisms and pathogenic bacteria, leading to a deterioration in quality during processing and storage [81]. Therefore, the use of additives is necessary to delay or inhibit these processes, being that those of natural origin are the most accepted today by consumers. This has led to the search for new sources of preservatives [82].

Kim, Cho, & Han (2013) evaluated the antioxidant and antimicrobial activity of the ethanolic extracts of ten leafy green vegetables commonly consumed in East Asia. Among them, bok choy (*Brassica campestris* L. ssp. *chinensis*), chamnamul (*Pimpinella brachycarpa* (Kom.) Nakai), Chinese chives (*Allium tuberosum* Rottler ex Spreng), and pumpkin (*Curcubita moschata* Duch.) stood out. The leaves of these vegetables showed TPCs of 18.93, 23.65, 27.19, and 12.41 mg GAE/g, respectively. Chamnamul displayed the highest values of ABTS cation scavenging activity (63.53, 30.63, 23.24, and 18.23 mg TE/g sample for chamnamul, chives, bok choy, and pumpkin leaves, respectively). Moreover, the extracts of chamnamul displayed broad-range antimicrobial activity, so they could be effective against both Gram-positive and Gram-negative bacteria. Therefore, chamnamul leaf extracts were selected to be applied to raw beef patties. As expected, the application of chamnamul reduced the microbial load (TVC, LAB, coliform, and yeast and mold counts) during refrigerated storage. However, only coliform counts were significantly reduced (3.71 and 4.18 vs. 4.64 log CFU/g meat for 0.5% and 0.1% chamnamul extracts and control, respectively) at 12 days of storage. α-selinene, β-pinene, myrcene, γ-terpinene, and β-caryophyllene would be responsible for this antimicrobial activity [83]. Chamnamul extracts also had a positive effect on the lipid stability of beef patties during storage, delaying TBARS values (0.47 and 1.11 vs. 1.40 mg MDA/kg for 0.5% and 0.1% chamnamul extracts and negative controls, respectively). Within color parameters, treated samples showed a* values much lower than those presented in the control samples, which would be related to the presence of a green pigment in the extracts. However, the antioxidant compounds contained in the extract allowed for delayed metmyoglobin formation when it was incorporated at 0.1%. A sensory study would be necessary to confirm the overall acceptability of the product.

The polyphenolic richness of cauliflower (*Brassica oleraceae*) leaves gives them important antioxidant and antimicrobial properties. Their ethanolic extracts are characterized by high TPC (5439.75 mg GAE/100 g) and DPPH radical-scavenging activity (89.76%). This makes them an effective strategy to control oxidation in meat products. In this regard, Zhang et al. [74] studied the effect of cauliflower leaves on the stability of pork patties during refrigerated storage. The ethanolic extract obtained from the dried leaves was incorporated into pork patties at 2.5, 5, and 10 g/kg. As a result, microbial growth (TVC, LAB, and *Pseudomonas*) was reduced in patties containing cauliflower leaf extract in a dose-dependent pattern. The presence of chlorogenic acid, ferulic acid, gallic acid, and catechin would be responsible for this behavior [84]. The lower TVC counts in the samples treated with the extract made it possible to extend their shelf life up to 7 days of storage. In contrast, control samples exceeded the limits allowed (>10^6^ CFU) after four days of storage. In the case of LAB and *Pseudomonas*, the extracts caused an inhibitory effect that was more marked in patties treated with 10 g/kg (5.16 and 4.09 log CFU/g for LAB and *Pseudomonas*, respectively).

The addition of cauliflower extracts enhanced the TPC (10.77 vs. 132.70 mg GAE/100 g for control and samples treated with 10 g/kg, respectively) and antioxidant activity (21.22% vs. 48.74% for untreated and samples treated with 10 g/kg, respectively) of pork patties, probably linked to the transference of BACs from the cauliflower extract to the patties. This would justify the longer shelf life of the cauliflower-enriched patties, which was reflected in color stability, lipid oxidation, and protein oxidation. Within color parameters, the addition of the extract had a protective effect. This was reflected in the a* values, which were slowly reduced during storage (17.8% after 3 days of storage in the control vs. 20.6% after 9 days in samples treated with 10 g/kg). An inversely proportional behavior was reflected in metmyoglobin (MetMb) content. After seven days of storage, the samples treated with 5 and 10 g/kg showed values below the threshold perceptible by the consumer (MetMb > 40%) compared to those found in control samples (≅40% after 5 days of storage). The antioxidant activity of cauliflower leaf extract was also reflected in TBARS values, which were lower in treated samples in a dose-dependent pattern. The results showed that it could be used as an alternative to synthetic antioxidants, since it even improved the values obtained for BHA (0.95 vs. 0.81 and 0.73 for BHA and samples treated with 5 g/kg and 10 g/kg, respectively). In the same way, the addition of the extract inhibited protein oxidation (5.74, 5.00, and 4.12 nmol/mg protein for controls and samples treated with BHA and 10 g/kg, respectively). Finally, the protection effects of cauliflower leaf extracts on product quality were also reflected in the sensory attributes, which were not affected by the inclusion of the extract. Patties treated with the vegetable extract received higher scores than those obtained in the control. Moreover, the scores obtained for color (2.27, 2.90, 2.67 and 3.13 for control, and samples treated with 2.5, 5 and 10 g/kg, respectively), odor (2.23, 2.90, 2.73 and 3.10) and overall acceptability (2.13, 2.83, 2.63 and 3.00) were higher in samples treated with higher concentration.

Nowadays, lifestyles make ready-to-cook products more and more in demand. Croquettes are among these types of products. It is a food product made from chicken, fish, or meat along with potatoes and various types of seasonings. Its quality depends fundamentally on its formulation and the cooking method [76]. Ashfaq et al. [77] evaluated the effect of the incorporation of green or red cabbage (as shreds or aqueous extracts) on the antioxidant capacity and physico-sensory parameters of chicken croquettes. Moreover, the cooking effect (from baking and frying procedures) was also investigated. The results showed that treated samples had the highest antioxidant capacity, with red cabbage-based croquettes obtaining the highest values, most likely due to their higher total polyphenol and flavonoid contents. Regarding cooking methods, fried croquettes displayed higher total polyphenols than baked samples (71.17–125.82 vs. 70.59–121.61 mg GAE/100 g FW, respectively), while total flavonoids were higher in baked coquettes (64.88–92.83 vs. 51.84–78.17 mg QE/100 g FW for baked and fried samples, respectively). These differences could be due to the degradation that polyphenols undergo due to the heat treatment, the activation of oxidation processing, or leeching in the cooking medium. This would be the case with the lower presence of flavonoids in fried croquettes, which could be due to their leaching into the frying oil. Although the incorporation of cabbage displayed significant differences between the samples for some of the sensory attributes evaluated, especially in odor, in general it did not affect product acceptability. The maximum scores were obtained for fried and baked (7.71 and 7.27, respectively) green cabbage-based croquettes. Similar results were observed in other meat products where cabbage was added as a natural antioxidant or source of dietary fiber for the development of functional products [75,76].

Verma et al. [75] studied the possibility of replacing meat with green cabbage (15 and 25%) in mutton patties. As expected, the incorporation of cabbage decreased fat contents, especially in those with a greater fat substitution (13.09, 14.35, and 16.55% for meat replacements of 15% and 25% and control samples, respectively). Regarding fat stability during storage, treated samples had a lower content of free fatty acids (FFA) as compared to the control (0.70 and 0.71 vs. 0.82% oleic acid for meat replacements of 15% and 25% and control samples, respectively). The same trend was observed in TBARS values and microbial counts. This would be related to green cabbage’s antioxidant properties associated with polyphenols and flavonoids, as well as its content of glucosinolates (allyl-isothiocyanate), which is considered one of the most important phytochemicals with antioxidant and antimicrobial properties. Along with these, isothiocyanates (hydrolysis products of glucosinolates) and methyl methanethiosulfinate would be responsible for microbial inactivation. Moreover, the incorporation of cabbage in chicken meatballs allowed a better maintenance of sensory attributes, with the samples treated with 15% green cabbage being the most suitable compared to other levels (6.89, 6.17, and 5.94 for the overall acceptability of samples in which 15% and 25% of the meat was substituted, and control samples, respectively).

The incorporation of cabbage powder also improved the quality of mutton patties, enhancing their nutritional properties and extending their shelf life [76]. The powder, characterized by TPC of 3.22 mg tannic acid (TA) equivalents/g DM, and 41.89% DPPH radical-scavenging activity, was added at three levels (6, 9 and 12%). In patties, the increase in the level of cabbage powder resulted in lower sensory scores, while levels of 6% showed values similar to the control (7.21, 7.08, 6.89, and 6.61 for the control, and cabbage powder levels of 6, 9, and 12%, respectively). The addition of 6% cabbage powder increased TPC (7.34 vs. 1.39 mg TA eq/g DM), and antimicrobial activity (22.51 vs. 5.76%) of patties compared to control. This was reflected in the values found for TBARS, which were lower in samples treated with cabbage both in aerobic storage (0.49 vs. 0.71 mg MDA/kg at 21 days of storage) and vacuum packaging (0.61 vs. 0.86 mg MDA/kg at 60 days of storage). The same results were observed for FFA (0.95 vs. 0.71% and 0.98 vs. 0.83% for control and treated samples in aerobic and vacuum packaging, respectively). Color also reflected the positive effects of the natural antioxidants contained in cabbage, since allowed to reduce the loss of color during storage (7.11 vs. 6.13 for treated and control samples in aerobic packaging, respectively). This is especially important for consumers since a decrease in redness could reduce the acceptability of the product. In addition, cabbage significantly reduced the microbial counts of mutton patties. 

Kimchi, a traditional Korean fermented food made of various vegetables, is also a good source of phenolic compounds. Lee et al. [78] evaluated the effect of various kimchi ethanolic extracts: baechu (Chinese cabbage with red pepper—T-BKE), got (mustard leaf, *Brassica juncea*—T-GKE), puchu (scallion, *Allium fistulosum*—T-PKE), and white kimchi (Chinese cabbage without red pepper—T-WKE) on the oxidative stability of raw ground pork meat during refrigerated storage. The incorporation of these extracts (1 g/kg) resulted in higher a* values than those obtained in control samples. This was also reflected in the total color difference (ΔE), where treated samples displayed the lowest values. Moreover, kimchi treatments were very useful in the inhibition of MetMb formation, with T-BKE extracts showing the lowest changes (40.79 vs. 66.99 for T-BKE and control, respectively). Regarding lipid oxidation, the values obtained were below the acceptability threshold (0.6 mg MDA/kg) for the rancid flavor in meat products, with GKE and PKE being the most effective extracts. Therefore, the use of kimchi extracts, especially GKE, would be a good strategy to reduce the color degradation, lipid oxidation, and bacterial counts of raw ground pork meat.

In addition to the antioxidant and antimicrobial effects of leafy vegetables, they can also be used as a natural source of nitrates. Their use would avoid the abuse of synthetic nitrates and their harmful effects on human health. In addition, their incorporation into meat products would also allow the production of clean-label products, which would meet consumer demands for healthy products. Among these leafy vegetables, beet, lettuce, arugula, spinach, chard, celery, and watercress stand out for their high nitrate contents [85,86]. In this regard, Martínez et al. [71] produced Spanish chorizo without synthetic additives. These preservatives were replaced by three blends of green leafy vegetables: LAW (lettuce, arugula, and watercress), SCe (spinach, celery), and ChB (chard, beet), which were combined with citric acid or rosemary to extend the shelf life of the fermented product. Previously, the authors described the natural extracts. Watercress (334.7 mg GAE/100 g), arugula (296.3 mg GAE/100 g), and chard (278.0 mg GAE/100 g) were the ones with the highest TPC, while beet (1384.1 ppm NO3^−^), chard (1213.4 ppm NO3^−^), and arugula (1160.5 ppm NO3^−^) presented a higher concentration of nitrates. In terms of antioxidant activity, beet was the vegetable with the highest activity measured as ABTS (85.7%), DPPH (90.2%), ORAC (3509.0 µM TE/g) and FRAP (3690 µM TE/g), followed by watercress (33.4% and 2510.3 µM TE/g for ABTS and FRAP, respectively). Moreover, the extracts had an excellent antimicrobial activity against *Clostridium perfringens*, which would be related to their total phenolic and nitrate contents. The incorporation into the product showed that the addition of these natural antioxidants reduced the volatile compounds from lipid oxidation (propan-2-ol, hexanal, and nonanal), and the microbial counts after 50 days of refrigerated storage. Quercetin, kaempferol, apigenin, ferulic acid, caffeic acid, and ρ-coumaric acid would be responsible for the activity of these extracts, which was increased with the addition of citric acid and rosemary.

### 3.5. Savoury Snacks

Current lifestyles are associated with an increase in the consumption of fast foods, such as snacks, which can be a good source of energy, fat, salt, and carbohydrates, but are not so good for your health. This makes a reformulation of these products necessary. Nazzaro et al. [87] evaluated the polyphenol composition and the antioxidant activity of four types of kale-based snacks (chips and crackers) common in the markets of Australia, Northern Europe, and the USA. The results showed that this product was a perfect matrix to deliver vegetal-derived phenolic antioxidants, representing one of the potential products for functional food markets. The polyphenol profile showed that gallic acid, chlorogenic acid, and catechin were the main polyphenols identified in the ethanolic extracts. Other compounds were also identified, although in lower quantities, as caffeic acid, r-coumaric acid, and ferulic acid. Therefore, the incorporation of kale in the snacks did not negatively affect the beneficial effect of the raw material.

## 4. Conclusions

Leafy vegetables are known for their high concentrations of bioactive compounds, the most notable of which are polyphenols. The identification of these compounds and their nutraceutical potential is very important since the trend within the food industry is to use natural ingredients to make their products. In this regard, the antioxidant activity associated with phenolic acids and flavonoids makes them good candidates to replace synthetic additives. Their application in bakery, meat, seafood, or fermented dairy products allows for control of the oxidative processes responsible for the quality deterioration of the products. Moreover, the microbiological properties and the high nitrate content of many of them delay or inhibit microbial growth, allowing for the production of a safe product. Therefore, the incorporation of these leaf vegetables, either in the form of extracts or powder, meets consumer expectations for a healthier product under the "clean label" concept. However, it is still necessary to optimize the levels at which they are incorporated to improve the sensory characteristics so that they are closer to those of the original product and, therefore, have greater acceptance.

## Figures and Tables

**Table 1:** Effect of natural extracts obtained from the leaves of different endemic plants on the shelf life of dairy products.

Leaf Vegetable	Dairy Product	Extract Dose	Storage Conditions	Main Results	Ref.
Celery (*Apium graveolens*)	White soft cheese	5, 10 and 15%	20 days at 4 °C	The incorporation of celery leaves had no significant differences in the proximate composition. Treated samples displayed lower total bacteria counts. Overall acceptability was not affected by the addition of celery.	[61]
*Allium roseum*	Double Cream Cheese	6% paste and 0.8% powder	15 days at 5 °C	Positive effects on sensorial, physicochemical, and microbiological qualities. Significant shelf life extension compared to control (12 vs. 10 days).	[62]
	Soft cheese made from ultrafiltered dromedary milk	0.5% (*w*/*w*)	4 °C	Improved the DPPH•-inhibition and the phenolic profile of the treated cheese without impairing the sensory quality.	[63]
Spinach (*Spinach oleracea*)	UF-Soft cheese	0.50, 1.00, 1.50, and 2%	4 weeks at cold storage	The incorporation of spinach nano-powder increased the nutritional value of the cheese and improved its TPC and antioxidant activity. Small amounts of spinach leaf powder also enhanced the color and taste of these products.	[64]

## Data Availability

Not applicable.

## References

[1] Kadam D.M., Monika S., Devinder K. (2017). Vegetables Process and Bioactive Compounds.

[2] Barba F.J., Esteve M.J., Frígola A., Rahman A. (2014). Bioactive components from leaf vegetable products. Studies in Natural Products Chemistry.

[3] Mansoor S., Ali Wani O., Lone J.K., Manhas S., Kour N., Alam P., Ahmad A., Ahmad P. (2022). Reactive Oxygen Species in plants: From source to sink. Antioxidants.

[4] Velázquez L., Quiñones J., Díaz R., Pateiro M., Lorenzo J.M., Sepúlveda N. (2021). Natural Antioxidants from Endemic Leaves in the Elaboration of Processed Meat Products: Current Status. Antioxidants.

[5] Ramírez-Rojo M.I., Vargas-Sánchez R.D., del Mar Torres-Martínez B., Torrescano-Urrutia G.R., Lorenzo J.M., Sánchez-Escalante A. (2019). Inclusion of ethanol extract of mesquite leaves to enhance the oxidative stability of pork patties. Foods.

[6] Yalcin H., Çapar T.D., Yildiz F., Wiley R.C. (2017). Bioactive compounds of fruits and vegetables. Minimally Processed Refrigerated Fruits and Vegetables.

[7] Duma M., Alsina I., Zeipina S., Lepse L., Dubov L. Leaf Vegetables as Source of Phytochemicals. Proceedings of the 9th Baltic Conference on Food Science and Technology “Food for Consumer Well-Being”—FOODBALT.

[8] Blasi F., Urbani E., Cossignani L., Simonetti M.S., Chiesi C. (2016). Seasonal variations in antioxidant compounds of *Olea europaea* leaves collected from different Italian cultivars Food Safety and Food Quality View project Structured lipid View project Seasonal variations in antioxidant compounds of *Olea europaea* leaves collected from different Italian cultivars. Artic. J. Appl. Bot. Food Qual..

[9] Alirezalu K., Pateiro M., Yaghoubi M., Alirezalu A., Peighambardoust S.H., Lorenzo J.M. (2020). Phytochemical constituents, advanced extraction technologies and techno-functional properties of selected Mediterranean plants for use in meat products. A comprehensive review. Trends Food Sci. Technol..

[10] Goyeneche R., Roura S., Ponce A., Vega-Gálvez A., Quispe-Fuentes I., Uribe E., Di Scala K. (2015). Chemical characterization and antioxidant capacity of red radish (*Raphanus sativus* L.) leaves and roots. J. Funct. Foods.

[11] Singh V., Chauhan G., Krishan P., Shri R. (2018). *Allium schoenoprasum* L.: A review of phytochemistry, pharmacology and future directions. Nat. Prod. Res..

[12] Vlase L., Parvu M., Parvu E., Toiu A. (2012). Chemical Constituents of Three *Allium* Species from Romania. Molecules.

[13] Pellegrini M.C., Ponce A.G. (2020). Beet (*Beta vulgaris*) and Leek (*Allium porrum*) Leaves as a Source of Bioactive Compounds with Anti-quorum Sensing and Anti-biofilm Activity. Waste Biomass Valoriz..

[14] Beretta H.V., Bannoud F., Insani M., Berli F., Hirschegger P., Galmarini C.R., Cavagnaro P.F. (2017). Relationships between bioactive compound content and the antiplatelet and antioxidant activities of six *Allium* vegetable species. Food Technol. Biotechnol..

[15] Štajner D., Popović B.M., Ćalić-Dragosavac D., Malenčić Đ., Zdravković-Korać S. (2011). Comparative Study on *Allium schoenoprasum* Cultivated Plant and *Allium schoenoprasum* Tissue Culture Organs Antioxidant Status. Phyther. Res..

[16] Kucekova Z., Mlcek J., Humpolicek P., Rop O., Valasek P., Saha P. (2011). Phenolic Compounds from *Allium schoenoprasum*, *Tragopogon pratensis* and *Rumex acetosa* and Their Antiproliferative Effects. Molecules.

[17] Kulig D., Matysiak M., Baldovská S., Štefániková J., Maruniaková N., Mňahončáková E., Árvay J., Galbavý D., Kolesárová A. (2019). Screening of polyphenolic compounds from traditional medicinal herbs. J. Microbiol. Biotechnol. Food Sci..

[18] Liu G., Zhuang L., Song D., Lu C., Xu X. (2017). Isolation, purification, and identification of the main phenolic compounds from leaves of celery (*Apium graveolens* L. *var. dulce* Mill./Pers.). J. Sep. Sci..

[19] Kooti W., Daraei N. (2017). A Review of the Antioxidant Activity of Celery (*Apium graveolens* L). J. Evid. Based. Complementary Altern. Med..

[20] Materska M., Olszówka K., Chilczuk B., Stochmal A., Pecio Ł., Pacholczyk-Sienicka B., Piacente S., Pizza C., Masullo M. (2019). Polyphenolic profiles in lettuce (*Lactuca sativa* L.) after CaCl_2_ treatment and cold storage. Eur. Food Res. Technol..

[21] González-Romero J., Arranz-Arranz S., Verardo V., García-Villanova B., Guerra-Hernández E.J. (2020). Bioactive Compounds and Antioxidant Capacity of Moringa Leaves Grown in Spain Versus 28 Leaves Commonly Consumed in Pre-Packaged Salads. Processes.

[22] Viacava G.E., Roura S.I., López-Márquez D.M., Berrueta L.A., Gallo B., Alonso-Salces R.M. (2018). Polyphenolic profile of butterhead lettuce cultivar by ultrahigh performance liquid chromatography coupled online to UV–visible spectrophotometry and quadrupole time-of-flight mass spectrometry. Food Chem..

[23] Alarcón-Flores M.I., Romero-González R., Martínez Vidal J.L., Garrido Frenich A. (2016). Multiclass Determination of Phenolic Compounds in Different Varieties of Tomato and Lettuce by Ultra High Performance Liquid Chromatography Coupled to Tandem Mass Spectrometry. Int. J. Food Prop..

[24] Chihoub W., Dias M.I., Barros L., Calhelha R.C., Alves M.J., Harzallah-Skhiri F., Ferreira I.C.F.R. (2019). Valorisation of the green waste parts from turnip, radish and wild cardoon: Nutritional value, phenolic profile and bioactivity evaluation. Food Res. Int..

[25] Šamec D., Pavlović I., Salopek-Sondi B. (2017). White cabbage (*Brassica oleracea* var. *capitata* f. *alba*): Botanical, phytochemical and pharmacological overview. Phytochem. Rev..

[26] Olsen H., Aaby K., Borge G.I.A. (2009). Characterization and quantification of flavonoids and hydroxycinnamic acids in curly kale (*Brassica oleracea* L. convar. *acephala* var. *sabellica*) by HPLC-DAD-ESI-MS^n^. J. Agric. Food Chem..

[27] Kaulmann A., Jonville M.C., Schneider Y.J., Hoffmann L., Bohn T. (2014). Carotenoids, polyphenols and micronutrient profiles of *Brassica oleraceae* and plum varieties and their contribution to measures of total antioxidant capacity. Food Chem..

[28] Mageney V., Neugart S., Albach D. (2017). A Guide to the Variability of Flavonoids in *Brassica oleracea*. Molecules.

[29] Upadhyay R., Sehwag S., Singh S.P. (2016). Antioxidant Activity and Polyphenol Content of *Brassica oleracea* Varieties. Int. J. Veg. Sci..

[30] Koss-Mikołajczyk I., Kusznierewicz B., Wiczkowski W., Płatosz N., Bartoszek A. (2019). Phytochemical composition and biological activities of differently pigmented cabbage (*Brassica oleracea* var. *capitata*) and cauliflower (*Brassica oleracea* var. *botrytis*) varieties. J. Sci. Food Agric..

[31] Jaiswal A.K., Abu-Ghannam N., Gupta S. (2012). A comparative study on the polyphenolic content, antibacterial activity and antioxidant capacity of different solvent extracts of *Brassica oleracea* vegetables. Int. J. Food Sci. Technol..

[32] Abdel-Shafi S., Al-Mohammadi A.-R., Sitohy M., Mosa B., Ismaiel A., Enan G., Osman A. (2019). Antimicrobial Activity and Chemical Constitution of the Crude, Phenolic-Rich Extracts of *Hibiscus sabdariffa*, *Brassica oleracea* and *Beta vulgaris*. Molecules.

[33] Ben Sassi A., Cheikh M’hamed A., Chahdoura H., Saidani Tounsi M., Mastouri M., Ben Salem H. (2020). Variation in biochemical profile and health beneficial compounds and biological activities of *Brassica oleracea* var *gongylodes* L. morphological parts. J. Food Meas. Charact..

[34] Roby M.H.H., Sarhan M.A., Selim K.A.-H., Khalel K.I. (2013). Evaluation of antioxidant activity, total phenols and phenolic compounds in thyme (*Thymus vulgaris* L.), sage (*Salvia officinalis* L.), and marjoram (*Origanum majorana* L.) extracts. Ind. Crops Prod..

[35] Ben Sassi A., Ascrizzi R., Chiboub W., Cheikh Mhamed A., ElAyeb A., Skhiri F., Tounsi Saidani M., Mastouri M., Flamini G. (2021). Volatiles, phenolic compounds, antioxidant and antibacterial properties of kohlrabi leaves. Nat. Prod. Res..

[36] Šamec D., Urlić B., Salopek-Sondi B. (2019). Kale (*Brassica oleracea* var. *acephala*) as a superfood: Review of the scientific evidence behind the statement. Crit. Rev. Food Sci. Nutr..

[37] Armesto J., Carballo J., Martínez S. (2015). Physicochemical and phytochemical properties of two phenotypes of Galega kale (*Brassica oleracea* L. var. *acephala* cv. Galega). J. Food Biochem..

[38] Akdaş Z.Z., Bakkalbaşı E. (2017). Influence of different cooking methods on color, bioactive compounds, and antioxidant activity of kale. Int. J. Food Prop..

[39] Ayaz F.A., Hayirlioglu-Ayaz S., Alpay-Karaoglu S., Grúz J., Valentová K., Ulrichová J., Strnad M. (2008). Phenolic acid contents of kale (*Brassica oleraceae* L. var. *acephala* DC.) extracts and their antioxidant and antibacterial activities. Food Chem..

[40] Jeon J., Kim J.K., Kim H.R., Kim Y.J., Park Y.J., Kim S.J., Kim C., Park S.U. (2018). Transcriptome analysis and metabolic profiling of green and red kale (*Brassica oleracea* var. *acephala*) seedlings. Food Chem..

[41] Nićiforović N., Abramovič H. (2014). Sinapic Acid and Its Derivatives: Natural Sources and Bioactivity. Compr. Rev. Food Sci. Food Saf..

[42] Park C.H., Bong S.J., Lim C.J., Kim J.K., Park S.U. (2020). Transcriptome Analysis and Metabolic Profiling of Green and Red Mizuna (*Brassica rapa* L. var. *japonica*). Foods.

[43] Seong G.U., Hwang I.W., Chung S.K. (2016). Antioxidant capacities and polyphenolics of Chinese cabbage (*Brassica rapa* L. ssp. *Pekinensis*) leaves. Food Chem..

[44] Yeo H.J., Baek S.-A., Sathasivam R., Kim J.K., Park S.U. (2021). Metabolomic analysis reveals the interaction of primary and secondary metabolism in white, pale green, and green pak choi (*Brassica rapa* subsp. *chinensis*). Appl. Biol. Chem..

[45] Tsouvaltzis P., Brecht J.K. (2014). Changes in Quality and Antioxidant Enzyme Activities of Bunched and Topped Radish (*Raphanus sativus* L.) Plants during Storage at 5 or 10C. J. Food Qual..

[46] Goyeneche R., Fanovich A., Rodriguez Rodrigues C., Nicolao M.C., Di Scala K. (2018). Supercritical CO_2_ extraction of bioactive compounds from radish leaves: Yield, antioxidant capacity and cytotoxicity. J. Supercrit. Fluids.

[47] Mzoughi Z., Chahdoura H., Chakroun Y., Cámara M., Fernández-Ruiz V., Morales P., Mosbah H., Flamini G., Snoussi M., Majdoub H. (2019). Wild edible Swiss chard leaves (*Beta vulgaris* L. var. *cicla*): Nutritional, phytochemical composition and biological activities. Food Res. Int..

[48] Rocha M.I., Rodrigues M.J., Pereira C., Pereira H., da Silva M.M., da Neng N.R., Nogueira J.M.F., Varela J., Barreira L., Custódio L. (2017). Biochemical profile and *in vitro* neuroprotective properties of *Carpobrotus edulis* L., a medicinal and edible halophyte native to the coast of South Africa. S. Afr. J. Bot..

[49] Fernández M.V., Jagus R.J., Agüero M.V. (2017). Evaluation and characterization of nutritional, microbiological and sensory properties of beet greens. Acta Sci. Nutr. Health.

[50] Biondo P.B.F., Boeing J.S., Barizão É.O., de Souza N.E., Matsushita M., de Oliveira C.C., Boroski M., Visentainer J.V. (2014). Evaluation of beetroot (*Beta vulgaris* L.) leaves during its developmental stages: A chemical composition study. Food Sci. Technol..

[51] Soquetta M.B., Stefanello F.S., Huerta K.D.M., Monteiro S.S., Da Rosa C.S., Terra N.N. (2016). Characterization of physiochemical and microbiological properties, and bioactive compounds, of flour made from the skin and bagasse of kiwi fruit (*Actinidia deliciosa*). Food Chem..

[52] Munekata P.E.S., Rocchetti G., Pateiro M., Lucini L., Domínguez R., Lorenzo J.M. (2020). Addition of plant extracts to meat and meat products to extend shelf-life and health-promoting attributes: An overview. Curr. Opin. Food Sci..

[53] Carvalho J.C.S., Romoff P., Lannes S.C.D.S. (2018). Improvement of nutritional and physicochemical proprieties of milk chocolates enriched with kale (*Brassica olereacea* var. *acephala*) and grape (*Vitis vinífera*). Food Sci. Technol..

[54] Galla N.R., Pamidighantam P.R., Karakala B., Gurusiddaiah M.R., Akula S. (2017). Nutritional, textural and sensory quality of biscuits supplemented with spinach (*Spinacia oleracea* L.). Int. J. Gastron. Food Sci..

[55] Drabińska N., Ciska E., Szmatowicz B., Krupa-Kozak U. (2018). Broccoli by-products improve the nutraceutical potential of gluten-free mini sponge cakes. Food Chem..

[56] Domínguez-Perles R., Martínez-Ballesta M.C., Carvajal M., García-Viguera C., Moreno D.A. (2010). Broccoli-Derived By-Products-A Promising Source of Bioactive Ingredients. J. Food Sci..

[57] Hwang J.H., Lim S. (2015). Bin Antioxidant and anticancer activities of broccoli by-products from different cultivars and maturity stages at harvest. Prev. Nutr. Food Sci..

[58] Ribeiro T.C., Abreu J.P., Freitas M.C.J., Pumar M., Teodoro A.J. (2015). Substitution of wheat flour with cauliflower flour in bakery products: Effects on chemical, physical, antioxidant properties and sensory analyses. Int. Food Res. J..

[59] D’Amico D.J., Donnelly C.W., McSweeney P.L.H., Fox P.F., Cotter P.D., Everett D.W. (2017). Growth and survival of microbial pathogens in cheese. Cheese: Chemistry, Physics and Microbiology.

[60] Ritota M., Manzi P. (2020). Natural Preservatives from Plant in Cheese Making. Animals.

[61] Awda J.M., Awad H.A., Alssirag M.A., Alfalahi D.A. (2019). Extend the shelf life and improving sensory properties of white soft cheese by adding celery leaves. Iraqi J. Agric. Sci..

[62] Gliguem H., Ben Hassine D., Ben Haj Said L., Ben Tekaya I., Rahmani R., Bellagha S. (2021). Supplementation of Double Cream Cheese with *Allium roseum*: Effects on Quality Improvement and Shelf-Life Extension. Foods.

[63] El Hatmi H., Jrad Z., Mkadem W., Chahbani A., Oussaief O., Zid M.B., Nouha M., Zaidi S., Khorchani S., Belguith K. (2020). Fortification of soft cheese made from ultrafiltered dromedary milk with *Allium roseum* powder: Effects on textural, radical scavenging, phenolic profile and sensory characteristics. LWT.

[64] El-Sayed S.M. (2020). Use of spinach powder as functional ingredient in the manufacture of UF-Soft cheese. Heliyon.

[65] Bozin B., Mimica-Dukic N., Samojlik I., Goran A., Igic R. (2008). Phenolics as antioxidants in garlic (*Allium sativum* L., Alliaceae). Food Chem..

[66] Pârvu M., Pârvu A.E., Vlase L., Rosca-Casian O., Pârvu O. (2011). Antifungal properties of *Allium ursinum* L. ethanol extract. J. Med. Plants Res..

[67] Dziri S., Hassen I., Fatnassi S., Mrabet Y., Casabianca H., Hanchi B., Hosni K. (2012). Phenolic constituents, antioxidant and antimicrobial activities of rosy garlic (*Allium roseum* var. *odoratissimum*). J. Funct. Foods.

[68] Jebelli Javan A., Bolandi M., Jadidi Z., Parsaeimehr M., Javaheri Vayeghan A. (2015). Effects of Scrophularia striata water extract on quality and shelf life of rainbow trout (*Oncorhynchus mykiss*) fillets during superchilled storage. Iran. J. Vet. Res..

[69] Raeisi S., Ojagh S.M., Sharifi-Rad M., Sharifi-Rad J., Quek S.Y. (2017). Evaluation of *Allium paradoxum* (M.B.) G. Don. and *Eryngium caucasicum* trauve. Extracts on the shelf-life and quality of silver carp (*Hypophthalmichthys molitrix*) fillets during refrigerated storage. J. Food Saf..

[70] Corzo-Martínez M., Corzo N., Villamiel M. (2007). Biological properties of onions and garlic. Trends Food Sci. Technol..

[71] Martínez L., Bastida P., Castillo J., Ros G., Nieto G. (2019). Green alternatives to synthetic antioxidants, antimicrobials, nitrates, and nitrites in clean label Spanish chorizo. Antioxidants.

[72] Kim S.J., Cho A.R., Han J. (2013). Antioxidant and antimicrobial activities of leafy green vegetable extracts and their applications to meat product preservation. Food Control.

[73] Aamer R.A., Emara H.H. (2016). Effect of Using Cauliflower (*Brassica oleracea*) to Improve Quality Characteristics of Tuna Fish Burger. Alex. J. Agric. Sci..

[74] Zhang H., Liang Y., Li X., Kang H. (2020). Antioxidant extract from cauliflower leaves effectively improve the stability of pork patties during refrigerated storage. J. Food Process. Preserv..

[75] Verma A.K., Pathak V., Singh V.P., Umaraw P. (2016). Storage study of chicken meatballs incorporated with green cabbage (*Brassica olerecea*) at refrigeration temperature (4 ± 1 °C) under aerobic packaging. J. Appl. Anim. Res..

[76] Malav O.P., Sharma B.D., Kumar R.R., Talukder S., Ahmed S.R., Irshad A. (2015). Antioxidant potential and quality characteristics of functional mutton patties incorporated with cabbage powder. Nutr. Food Sci..

[77] Ashfaq F., Butt M.S., Bilal A., Tehseen S., Suleria H.A.R. (2020). Effect of cabbage or its aqueous extract incorporated croquettes on chemical composition and storage stability in relation to antioxidant potential and sensory profile. J. Food Process. Preserv..

[78] Lee M., Kim T., Hwang K., Choi Y., Park S., Kim C., Choi Y. (2019). Kimchi extracts as inhibitors of colour deterioration and lipid oxidation in raw ground pork meat during refrigerated storage. J. Sci. Food Agric..

[79] Demirbaş A. (2020). Red Cabbage Extracts as Inhibitors of Lipid Oxidation in Fresh Minced Tilapia (*Nile perch*) During Refrigerated Storage. Turk. J. Agric. Food Sci. Technol..

[80] Barakat H., Rohn S. (2014). Effect of different cooking methods on bioactive compounds in vegetarian, broccoli-based bars. J. Funct. Foods.

[81] Lorenzo J.M., Vargas F.C., Strozzi I., Pateiro M., Furtado M.M., Sant’Ana A.S., Rocchetti G., Barba F.J., Dominguez R., Lucini L. (2018). Influence of pitanga leaf extracts on lipid and protein oxidation of pork burger during shelf-life. Food Res. Int..

[82] Pateiro M., Munekata P.E.S., Sant’Ana A.S., Domínguez R., Rodríguez-Lázaro D., Lorenzo J.M. (2021). Application of essential oils as antimicrobial agents against spoilage and pathogenic microorganisms in meat products. Int. J. Food Microbiol..

[83] Chang K.M., Chung M.S., Kim M.K., Kim G.H. (2007). Analysis of mineral and volatile flavor compounds in *Pimpinella brachycarpa* N. by ICP-AES and SDE, HS-SPME-GC/MS. J. Korean Soc. Food Cult..

[84] Cartea M.E., Francisco M., Soengas P., Velasco P. (2011). Phenolic compounds in *Brassica* vegetables. Molecules.

[85] Bahadoran Z., Mirmiran P., Jeddi S., Azizi F., Ghasemi A., Hadaegh F. (2016). Nitrate and nitrite content of vegetables, fruits, grains, legumes, dairy products, meats and processed meats. J. Food Compos. Anal..

[86] Munekata P.E.S., Pateiro M., Domínguez R., Pollonio M.A.R., Sepúlveda N., Andres S.C., Reyes J., Santos E.M., Lorenzo J.M. (2021). *Beta vulgaris* as a Natural Nitrate Source for Meat Products: A Review. Foods.

[87] Nazzaro F., Cardinale F., Cozzolino A., Granese T., Fratianni F. (2014). Polyphenol Composition and Antioxidant Activity of Different Potentially Functional Kale-Based Snacks. Food Nutr. Sci..

